# The Accelerating Exposure of European Protected Areas to Climate Change

**DOI:** 10.1111/gcb.70261

**Published:** 2025-06-06

**Authors:** Marta Cimatti, Valerio Mezzanotte, Risto K. Heikkinen, Maria H. Hällfors, Dirk Nikolaus Karger, Moreno Di Marco

**Affiliations:** ^1^ Department of Biology and Biotechnologies “Charles Darwin” Sapienza University of Rome Rome Italy; ^2^ Nature Solutions Finnish Environment Institute Helsinki Finland; ^3^ Swiss Federal Research Institute WSL Birmensdorf Switzerland

**Keywords:** climate change magnitude, climate change velocity, climate resilience, Natura 2000, protected area, Trans‐European Nature Network

## Abstract

All ecosystems are affected by climate change, but differences in the pace of change will render some areas more exposed than others. Such spatial patterns of risk are important when assessing the continued functionality of protected area (PA) networks or planning for their expansion. Europe is undertaking an expansion of the PA network to cover 30% of its land and sea surface by 2030, but this must account for climate risk. Here, we estimate four metrics of future climate risk across Europe: local velocity, analog velocity, magnitude, and residence time, and assess the level of climate exposure of European PAs vs. nonprotected control sites. We also evaluate the intensity of climate risks on > 1000 European species of conservation concern associated with Natura 2000 sites. Our results show large spatial differences in climate change exposure across Europe, with a faster pace and farther shifts in the Boreal, Steppic, and Pannonian regions but slower changes in the Mediterranean, Alpine, Arctic, and Macaronesia regions. The magnitude of climate change was higher for the Arctic, Alpine, and Mediterranean regions, implying large local differences between present and future climate. These spatial risk patterns were largely consistent across scenarios, but with up to three times higher risk under the most pessimistic vs. the most optimistic scenario. Large variation in climate exposure for species of conservation concern was revealed, including 11 species that are highly dependent on Natura 2000 sites and predicted to experience rapid climate change. Our results provide guidance for managing European PAs and expanding their coverage by pinpointing areas offering more stable climates. We emphasize the need for connectivity across the network to support species adaptation via range shifting. This is especially the case in areas facing high climate change magnitude but low velocity, implying that climate conditions similar to current ones will be found nearby.

## Graphical abstract

We assessed future climate risk for European protected areas, using four climate metrics. We predict high magnitude of change in the Arctic, Alpine, and Mediterranean regions, but high velocity of change in the Boreal, Steppic, and Pannonian regions. We found high risk for several Natura 2000 sites hosting species known to be sensitive to climate change. Our findings highlight the need for including climate adaptation strategies as part of any protected area expansion plan.

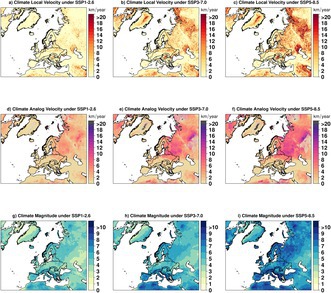

## 1 Introduction

Biodiversity and ecosystems support human societies worldwide, but the exploitation of natural resources and the conversion of natural land for human use is causing widespread biodiversity loss (Díaz et al. 2019; Dirzo et al. 2014). On top of these direct pressures, climate change is projected to cause accelerating impacts on biodiversity (Dawson 2011; Díaz et al. 2019; Urban 2015; Wiens and Zelinka 2024), intensifying the impacts of land‐use change (Brook et al. 2008; Heikkinen et al. 2021; Mantyka‐Pringle et al. 2012, 2015; Oliver et al. 2015). The primary means to halt the loss of biodiversity has been the establishment of protected areas (PAs) and other area‐based conservation interventions (Margules and Pressey 2000; Watson et al. 2014). PAs counteract (Santangeli et al. 2023) the overall loss of biodiversity and the fragmentation of ecosystems, and form the backbone of conservation strategies worldwide (S. Hoffmann et al. 2018; Pimm et al. 2014). Protection of areas also represent the only conservation mechanism to preserve the last remaining habitat of many rare and threatened species (Lawrence et al. 2021; Nila and Hossain 2019; Pacifici et al. 2020). The Kunming‐Montreal Global Biodiversity Framework calls for an expansion of “protected areas and other effective area‐based conservation measures—covering at least 30% of the planet by 2030”. In line with this framework, the EU Biodiversity Strategy commits member states to protect at least 30% of their land and sea surface by 2030, including 10% placed under strict protection.

In 2024, European PAs covered 26.1% of the land surface with the Natura 2000 network, the largest PA network in the world (EEA 2024; Eurostat 2022), covering 18.6% of the area. Natura 2000 offers substantial protection for species of conservation concern, including (but not limited to) those listed in the Birds Directive and Habitats Directive (Trochet and Schmeller 2013), although gaps in its coverage remain. In fact, many threatened mammals, birds, butterflies, and reptiles are substantially dependent on PAs and Natura 2000 sites for survival (van der Sluis et al. 2016; but see Ricci et al. 2024). However, European PAs, like most PA networks around the globe, have been established without proper consideration of their exposure to climate change (S. Hoffmann 2021; Lai et al. 2022). If PAs do not incorporate climate change into their design—which is a common shortcoming—their effectiveness in preserving biodiversity may become compromised (Araújo et al. 2011; Bellard et al. 2012; S. Hoffmann et al. 2019; Mendez Angarita et al. 2023).

Climate change does not always lead to species decline, as species may cope with new climatic conditions by altering their phenology, reproductive behavior, or ecology (Araújo et al. 2011; Bellard et al. 2012; S. Hoffmann et al. 2019; Mendez Angarita et al. 2023). When this is not possible, species need to shift their ranges to track their preferred climate. Terrestrial species are already moving poleward and/or toward higher elevations (Bellard et al. 2012; Hällfors et al. 2024; Scheffers et al. 2016; Thomas and Gillingham 2015), whereas aquatic species are moving deeper (Brito‐Morales et al. 2020; Jorda et al. 2020). These shifts pose challenges for biodiversity conservation, particularly when PAs do not incorporate climate change considerations into their initial design or management strategies. Without adaptive planning, species may be driven out of existing PAs, reducing their effectiveness (Lehikoinen et al. 2021; Loarie et al. 2009; Santangeli et al. 2017). To accommodate the needs of biodiversity under climate change, it is crucial to assess the projected climatic exposure of PAs and associated risks to species persistence (Lai et al. 2022).

In response to accelerating climate change, there is increased interest in assessing climate risks using metrics such as velocity and magnitude (Brito‐Morales et al. 2018; Garcia et al. 2014). Several global studies have analyzed the rate of predicted climate change in PAs (Elsen et al. 2020; S. Hoffmann et al. 2019; Loarie et al. 2009). A number of studies have also focused on climate risk in European terrestrial habitats and PAs (Araújo et al. 2011; Brito‐Morales et al. 2018; Heikkinen et al. 2020; Lai et al. 2022; Nila and Hossain 2019). Yet, despite the increased use of climate change metrics in PA studies, we still have an uncomprehensive understanding of how climatic risk can be measured through multiple metrics and across several climatic scenarios and models in PAs vs. control sites. Developing on this front would allow an improved understanding of the potential implications for species of conservation concern.

Here, we evaluate climate risk for Europe and its PAs at a 1 km resolution, using four metrics of climate change, three future emission scenarios, and three Earth System Models (ESM). We evaluate whether some regions are more highly exposed than others according to each metric, whether climate risks are higher inside vs. outside PAs, and finally, assess the level of risks for species of conservation concern. The four metrics of climate change used here represent complementary aspects of risk (Brito‐Morales et al. 2018; Loarie et al. 2009; Williams et al. 2007): (1) local‐based velocity, describing the intensity of change in a given location relative to local variation in climatic conditions; (2) analog velocity (distance‐based method), describing the distance between current climate in a location and the closest location with analog conditions in the future; (3) magnitude of change, depicting how much the climatic conditions of a location change over time; (4) residence time, which indicates how long current climatic conditions will persist within the boundaries of an area (in our case, a PA).

It is essential to use these metrics in combination as different regions, PAs, and species are differentially exposed to climate change and the risk that is measured by each metric describes different aspects of change (Garcia et al. 2014; Ordonez and Williams 2013). This assessment is thus a crucial step in climate‐proofing nature conservation initiatives, helping conservation planners to ensure the climatic resilience of existing and new PAs within the Trans‐European Nature Network (TEN‐N).

## 2 Materials and Methods

We examined the spatial variation of four complementary metrics of climatic exposure across Europe: local (gradient based) and analog (distance‐based method) climate velocity, magnitude of climate change, and residence time. For each metric, we compiled climate change data under nine combinations of three climate scenarios (SSP1‐2.6, SSP3‐7.0, SSP5‐8.5) and three ESMs (GFDL‐ESM4, UKESM1‐0‐LL, MPI‐ESM1‐2‐HR). This entailed a total of 36 climate risk predictions (4 metrics * 3 scenarios * 3 ESMs; Table S1). For each climate metric and climate scenario, we also generated an ensemble prediction across ESMs by averaging data from the three models and retrieved the standard deviation. Finally, we selected the first and last quartiles of each ensemble climatic metric, highlighting areas of relative low and high exposure, thereby identifying coldspot areas (i.e., climate refugia) where all metrics have a low value and hotspot areas where one or more metrics show high values. We calculated the percentage of PA, including areas of low and high risk.

**FIGURE 1:**
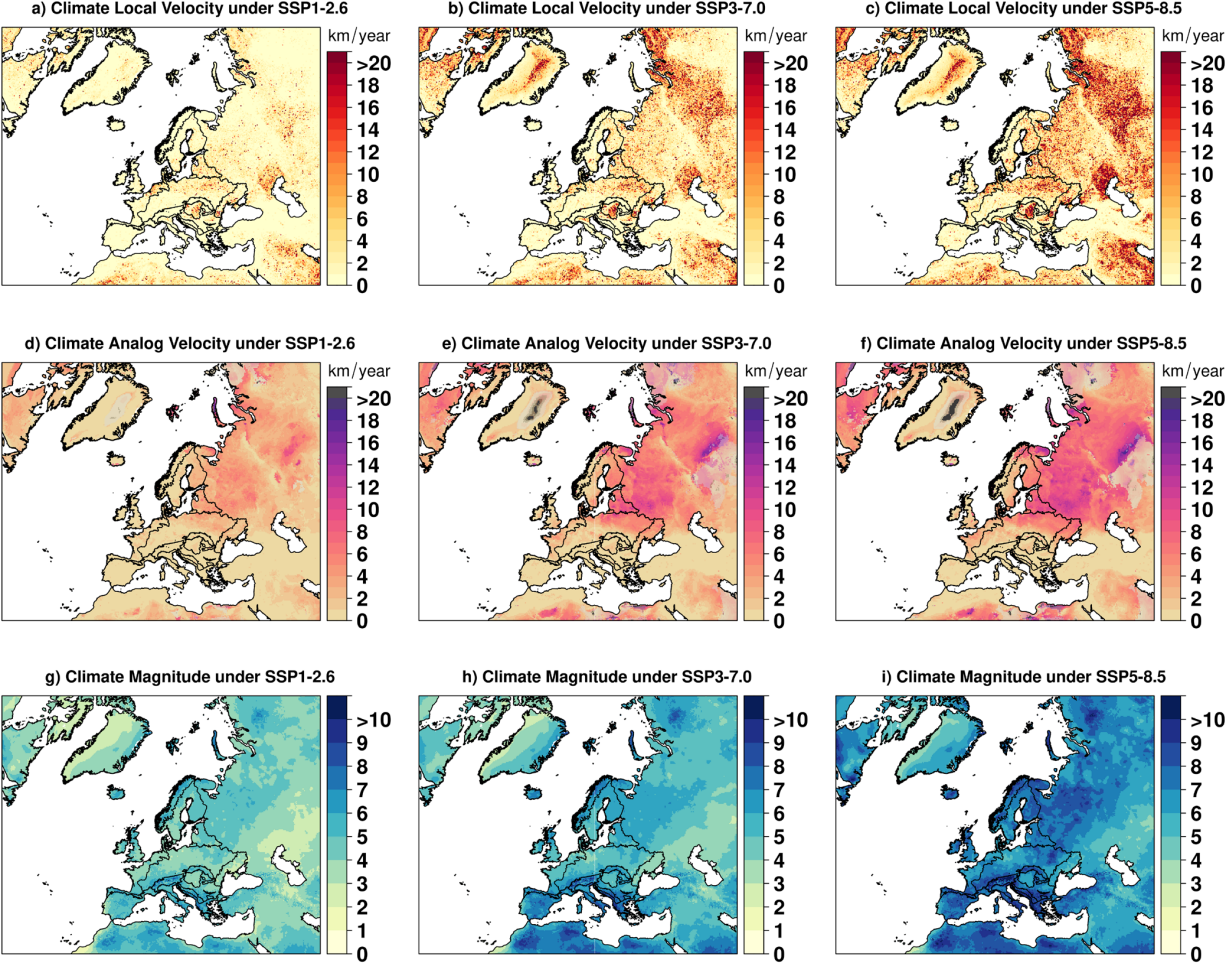
Estimates of different climatic metric values at 1 km resolution under scenarios SSP1‐2.6 (left‐hand panels), scenario SSP3‐7.0 (central panels), and scenario SSP5‐8.5 (right‐hand panels). Top‐row panels (a–c) show values for the absolute sum of local velocity. Mid‐row panels (d–f) show the climate analog velocity (distance‐based method), where areas in shaded colors depict raster cells for which there was no climate analog within the 1500 km buffer for one (light gray) or more (dark gray) ESM. Bottom panels (g–i) show values for climate magnitude (intensity of the change, adimensional). Metric values for individual climatic variables and ESM are shown in Figures S1–S3, S7, S8. Map lines delineate study areas and do not necessarily depict accepted national boundaries.

Our study area spans the territories of EU 46, extending from Greenland in the west to the Urals in the east, and including Turkey and parts of North African countries to encompass the entire Mediterranean shoreline (Figure 1 and Figures S1–S8). However, the study area used to identify control sites (via propensity score matching; see below) is narrower, as analyses related to PAs are focused on only states with exclusively European territories. It therefore includes the EU 28 countries, along with Belarus, Bosnia and Herzegovina, Moldova, Montenegro, North Macedonia, Norway, Serbia, Ukraine, and the United Kingdom, whose boundaries fall entirely within the European continent (Figure S4).

### 2.1 Selection of Climate Variables, Scenarios, and Models

We calculated climate change metrics based on two bioclimatic variables describing general climatic conditions: mean annual near‐surface (2 m) air‐temperature (*temperature*) and the annual precipitation rate (*precipitation*). We obtained yearly bioclimatic variables at 1 km^2^ resolution using the python package *chelsa_cmip6* (Karger et al. 2023, Appendix S1), and baseline and future climatologies from CHELSA V2.1 (Karger et al. 2017, 2021). We chose this reduced set of climate variables to represent overall climatic risk, in line with previous studies (Asamoah et al. 2022; Loarie et al. 2009). Using an ensemble of ESMs for different climate change scenarios is recommended for mastering the range of possible future climates and associated uncertainties (Pereira et al. 2010; Thuiller et al. 2019) stemming from different equilibrium climate sensitivity of ESMs (Knutti et al. 2017). We selected a set of scenarios from the Coupled Model Intercomparison Project Phase 6 (CMIP6) that encompass low to high levels of future carbon emission (O'Neill et al. 2016). In scenario SSP1‐2.6 “Sustainability—Taking the green road” an increasing shift toward sustainable practices is envisioned with persisting efforts to limit global warming to below 2°C compared to preindustrial level. Scenario SSP3‐7.0 “Regional Rivalry—A Rocky Road” involves strong land use change and entails the highest methane and air pollution precursor emissions with global mean temperature increases of 1.95°C–4.38°C by 2100 (Tebaldi et al. 2021). Scenario SSP5‐8.5 “Fossil‐fuelled Development—Taking the Highway” represents a world with a strong push for economic and social development at the expense of climate mitigation, with a temperature increase of 2.40°C–5.57°C by 2100 (Tebaldi et al. 2021). In parallel, we use three ESMs following the priority proposed by the Inter‐Sectoral Impact Model Intercomparison Project Phase 3 (ISIMIP) (Frieler et al. 2024; Lange 2019) to represent inherent uncertainty around climatic modeling approaches. This priority accounts for process representation, structural independence, equilibrium climate sensitivity, and model performance in the historical period. We selected these three models because they are structurally distinct in their oceanic and atmospheric components and encompass a range of process representations, from moderate (MPI‐ESM1‐2‐HR) to strong (GFDL‐ESM4, UKESM1‐0‐LL). Their climate sensitivity aligns with the broader CMIP6 ensemble, including models with lower sensitivity (GFDL‐ESM4, MPI‐ESM1‐2‐HR) and one with higher sensitivity (UKESM1‐0‐LL). This selection ensures a broad representation of potential climate responses, capturing the uncertainty inherent in future projections.

### 2.2 Selection of Climatic Metrics

We calculated four climatic metrics for each combination of climate scenarios and ESMs to quantify the exposure of PAs to climate change. The first metric, local velocity (*sensu* Loarie et al. 2009) is calculated based on local climatic gradients (Carroll et al. 2015; Loarie et al. 2009). This metric represents the rate of movement of climatic isopleths across a landscape (Loarie et al. 2009), describing the speed and direction at which a given organism needs to move to stay under similar climatic conditions. Local velocity provides estimates of species adaptation potential by finding suitable conditions adjacent to their current occurrence sites in subsequent years. We measured the local velocity of change following the gradient‐based approach (Loarie et al. 2009) and using an automated workflow based on the R functions *tempTrend* and *spatGrad* in the *VoCC* package (García Molinos et al. 2019). The velocity of change is defined as the ratio between the temporal trend of a climate variable, the rate of change of the variable through time for baseline (1981–2010) and future (2041–2070) periods (estimated as a regression slope on yearly data), and the corresponding local spatial gradient of that variable (i.e., the vector sum of longitudinal and latitudinal pairwise differences at each focal cell using a 3 × 3‐cell neighborhood), described in Equation (1).

(1) $$\text{Local Velocity}=\frac{\text{slope}\left(\frac{\text{unit}}{\text{year}}\right)}{\text{spatialgradient}\left(\frac{\text{unit}}{\mathrm{km}}\right)*}=\text{gVoCC}\left(\frac{\mathrm{km}}{\text{year}}\right)$$

*unit/year: (°C/km for temperature and mm/km for precipitation).

The second metric was the climate analog velocity (distance‐based method), which is based on geographical distances between locations of current climate classes and their future analogs (Brito‐Morales et al. 2018; Hamann et al. 2015). From an ecological perspective, this metric provides a measure of how far species need to move within a given time frame to find similar conditions to those currently experienced. Climate analog velocity is calculated as the distance, *d*, to the geographically closest climate analog for each focal cell divided by the time elapsed, *t*, between baseline (1981–2010) and future climate (2041–2070), calculated according to Equation (2).

(2) $$\text{Analog Velocity}=\frac{d}{t}=\text{dVocc}\left(\frac{\mathrm{km}}{\text{year}}\right)$$

Climate analogs were identified using multivariate (minimum) Euclidean distance between current and future climate, using a dissimilarity threshold of 1.5 standard deviations based on the variability of historical climate conditions (Doxa et al. 2022; García Molinos et al. 2017). Calculating velocity through analogs can pose computational challenges since it involves scouring the entire extent of the study area to find the spatially closest cell with similar climatic attributes of each focal cell (i.e., solving an *N* ^2^ problem, considering *N* = 29,632,989 cells). Thus, we limited the search radius around each focal cell to 1500 km, as a compromise between computational time and biological significance.

The third metric, climate change magnitude, measures change in a climate parameter over time at a given location, providing information on the extent of the change in each location in relative terms of the climate variables as they are standardized by historical interannual climate variability (Garcia et al. 2014). Ecologically, this metric provides direct information on the climate change to which a species is exposed in a given location (Williams et al. 2007; Williams and Jackson 2007). We quantified magnitude as dissimilarities between baseline (1981–2010) and future (2041–2070) climate by using the standardized Euclidean distance (SED) (Gavin et al. 2003; Williams et al. 2007), as in Equation (3).

(3) $$\text{Magnitude}=\sqrt{\sum_{k=1}^{2}\frac{{\left(b_{\mathrm{ki}}-a_{\mathrm{ki}}\right)}^{2}}{s_{\mathrm{ki}}^{2}}}={\mathrm{SED}}_{i}$$

In Equation (3) *a*_*ki*_ and *b*_*ki*_ are the baseline and future means for climate variable *k* at locations *i*, respectively, while $s_{\mathrm{ki}}^{2}$ is the standard deviation of the interannual variability for 1981–2010. The SED equally weights all variables and highlights predictions for 2041–2070 that are large relative to interannual variability during 1981–2010.

As our fourth metric, we assessed the climate residence time of European PAs. Residence time is a metric that describes after how long (e.g., in years) a specific isoline will cease to exist in a focal area (Brito‐Morales et al. 2018; Loarie et al. 2009). Ecologically, a short residence time means a given PA might soon become unable to sustain suitable conditions for the biodiversity it currently hosts (Heikkinen et al. 2020). In our analysis, we measured residence time as the time after which future climatic conditions disappear from within the PA. We calculated the residence time based on temperature and precipitation using the *VoCC* package and the *resTime* function (García Molinos et al. 2019), according to Equation (4):

(4) $$\text{Residence Time}=\left|\frac{d_{i}}{{\mathrm{gV}}_{i}}\right|\approx \left|\frac{2*\sqrt{\frac{A_{i}}{\pi }}}{{\mathrm{gV}}_{i}}\right|\left(\text{years}\right)$$

where *di* is the diameter of a PA's polygon *i*, which is calculated based on the areal size of the polygon (*Ai*), and *gVi* is the average climate velocity measured within the PA polygon *i*.

### 2.3 Comparison of Climatic Risk Inside vs. Outside PAs: Identification of Climatic Coldspots and Hotspots

We created a spatial map of European PAs by extracting data from the World Database on Protected Areas (WDPA, IUCN and UNEP‐WCMC 2023). The database contains multiple duplicates of the same areas (Vimal et al. 2021), one for each legislative instrument protecting them. To remove such duplicates and eliminate overlapping polygons, we used the *wdpa_clean* function in the R package *wdpar* (Hanson 2022) and assigned each PA to the highest IUCN protection category among those covered by several legislative instruments. We also generated circular buffers with areas matching the reported size around PAs that were listed as points but lacked polygonal boundaries. We included in our analysis only terrestrial sites, clipping PAs by the coast using the country borders' polygons from the Global Administrative Areas Dataset (GADM, https://gadm.org/). We defined a cell as “protected” (i.e., a subject receiving the treatment) if it overlapped by > 50% with a PA, and a cell as “nonprotected” (i.e., a control subject) if it overlapped by < 5% with a PA. Cells with marginal protection (between 5% and 50%) were excluded from the analysis.

We extracted median values of each climatic metric for each PA polygon and compared them with values in nonprotected “control” sites using a propensity score matching approach (Geldmann et al. 2019; Negret et al. 2020; Stuart et al. 2013). Propensity score matching is defined as the probability of receiving treatment, given specific covariates, which allows unbiased matching between subjects in the control condition and subjects who have “undergone treatment” (Olmos and Govindasamy 2015; Stuart et al. 2013). For every protected focal cell (i.e., our “subjects”), the propensity score matching selects a nonprotected cell with the most similar baseline environmental characteristics to provide an unbiased correspondence for comparison. We used several environmental variables in the matching process (Table S2): the 19 bioclimatic variables from CHELSA V.2.1 (Karger et al. 2017, 2021), land cover from Copernicus (Buchhorn et al. 2020), Human Footprint (Venter et al. 2016), and topography derived from the SRTM elevation data (Farr et al. 2007). Before performing the matching, all variables were standardized using the scale function in R (R Core Team 2024). Analyses were run at a 1 km^2^ resolution, reprojecting all the variables in ETRS89 Lambert Azimuthal Equal Area (ETRS_LAEA).

Matching was performed individually for each European biogeographical region (only including states with exclusively European territories) using the *MatchIt* package in R (Ho et al. 2011), and the nearest neighbor method without replacement. A threshold of SD < 0.25 was used to exclude low‐quality matches, where the distance in propensity scores between treatment and control was too low (Cuenca et al. 2016; Negret et al. 2020).

In order to assess the quality of matching, we checked for covariate balance before and after the matching within the original dataset and in the matched sample. To assess the balance between the treated and control groups, we used the standardized mean difference test (SMD) and the *C*‐statistic test. These tests allow comparing the distribution of baseline covariates between treatment groups in observational studies and as a balance measure of individual covariates before and after matching (Rahman and Islam 2021). Here, we used the *Cobalt* package (Greifer 2024), and set a cutoff at a standardized difference of 10%. SMDs close to zero indicate a good balance (Austin and Austin 2009; Haukoos and Lewis 2015; Rahman and Islam 2021), with recommended values of SMDs being below 0.1. The C‐statistic ranges from 0.5 to 1.0, with the minimum indicating that the propensity score model is not able to discriminate between treated and untreated units after matching, indicating a perfect balance of covariates (Franklin et al. 2014). Once matching was performed, we used a Wilcoxon test to assess whether significant differences existed between the climate change metrics of protected and unprotected areas in each biogeographic region (exact matching).

### 2.4 Climatic Exposure for Species Within Natura 2000 Areas

We estimated climate change exposure for species occurring in the Natura 2000 sites to assess the potential biodiversity implications within this PA network. We produced a list of species that meet two criteria: they are (I) identified as vulnerable to climate change according to the Threat Classification Scheme of the IUCN Red List (IUCN 2023) and (II) they are reported to occur within one or more Natura 2000 sites. In this case, using Natura 2000 sites, instead of all PAs, allowed us to select all species with verified presence in each site (information which is not systematically available for other PAs). This resulted in 1011 species encompassing vertebrates, invertebrates, and plants (Appendix S2). We measured the median values for each climate metric for all Natura 2000 sites in which at least one such species occurred.

For 514 species with detailed spatial distribution information available from the IUCN Red List, we also measured the percentage of overlap of the distribution with the Natura 2000 network. This allowed us to calculate the climatic metrics weighted according to the size of the PAs. Our aim was not to measure climate “risk” separately for each species, which would often require consideration of other factors such as species' sensitivity and adaptability (Foden et al. 2013). Instead, we use this to demonstrate the level of climate exposure that already vulnerable species are projected to face within protected sites.

## 3 Results

Overall, we found notable spatial differences in climate change exposure across Europe for all four climatic metrics considered, as well as among the three climate scenarios (Figure 1 and Figures S1–S3). We found that, under scenario SSP1‐2.6, the European median local (gradient based) climate velocity would decrease from a present (1981–2010) value of 1.77 km/year across the study area (Figure S5) to a future value of 0.73 km/year (Figure 1, SD = 1.95 km/year, Figure S6a). The situation was inverse under a high‐emission scenario, with a median future velocity of 3.22 km/year under SSP5‐8.5 (SD = 3.17 km/year, Figure 1 and Figure S1). Climate analog velocity (i.e., distance to analog climate between 1981–2010 and 2041–2070) increased in high‐emission scenario SSP5–8.5 (median value of 3.25 km/year, SD = 2.92 km/year, Figure 1 and Figure S2) compared to scenario SSP1‐2.6 (1.38 km/year, Figure 1 and Figure S2, SD = 1.71 km/year, Figure S6b). However, for the climate analog velocity, it was not always possible to find an analog climate within the evaluated 1500 km buffer around the focal cell. In particular, under the most extreme ESM (UKESM1‐0‐LL) and the most pessimistic emission scenario, large portions of the study area (up to 12.6% of the area under SSP5‐8.5) had no future analog within 1500 km (especially in the north‐eastern parts; Figure S2i). For this reason, our median climate analog velocity values for the whole study area can be considered an underestimation, while the results involving the EU 28 area are acceptable since only 0.66% of the cells had no future climatic analogs. The median climate magnitude (i.e., dissimilarity between 1981–2010 and 2041–2070 climates) showed similar trends as velocity metrics, ranging from 3.90 under scenario SSP1‐2.6 (Figure 1 and Figure S3, SD = 2.49, Figure S6c) to 6.13 (SD = 2.61) under scenario SSP5–8.5 (Figure 1 and Figure S3).

The most exposed biogeographic regions (highest median value before propensity score matching) were the Pannonian, Boreal, and Steppic for local velocity, while for climate analog velocity the most exposed regions were the Steppic, Boreal, and Arctic. For both velocity measures, the less exposed biogeographic regions were the Alpine, Mediterranean, and Macaronesia. In contrast, the Alpine and Mediterranean regions experienced the highest values for climate magnitude, followed by the Arctic, whereas the lowest magnitude values occurred in the Pannonian (Table S4).

### 3.1 Future Hotspots and Coldspots of Climatic Exposure and Their Protection

**FIGURE 2:**
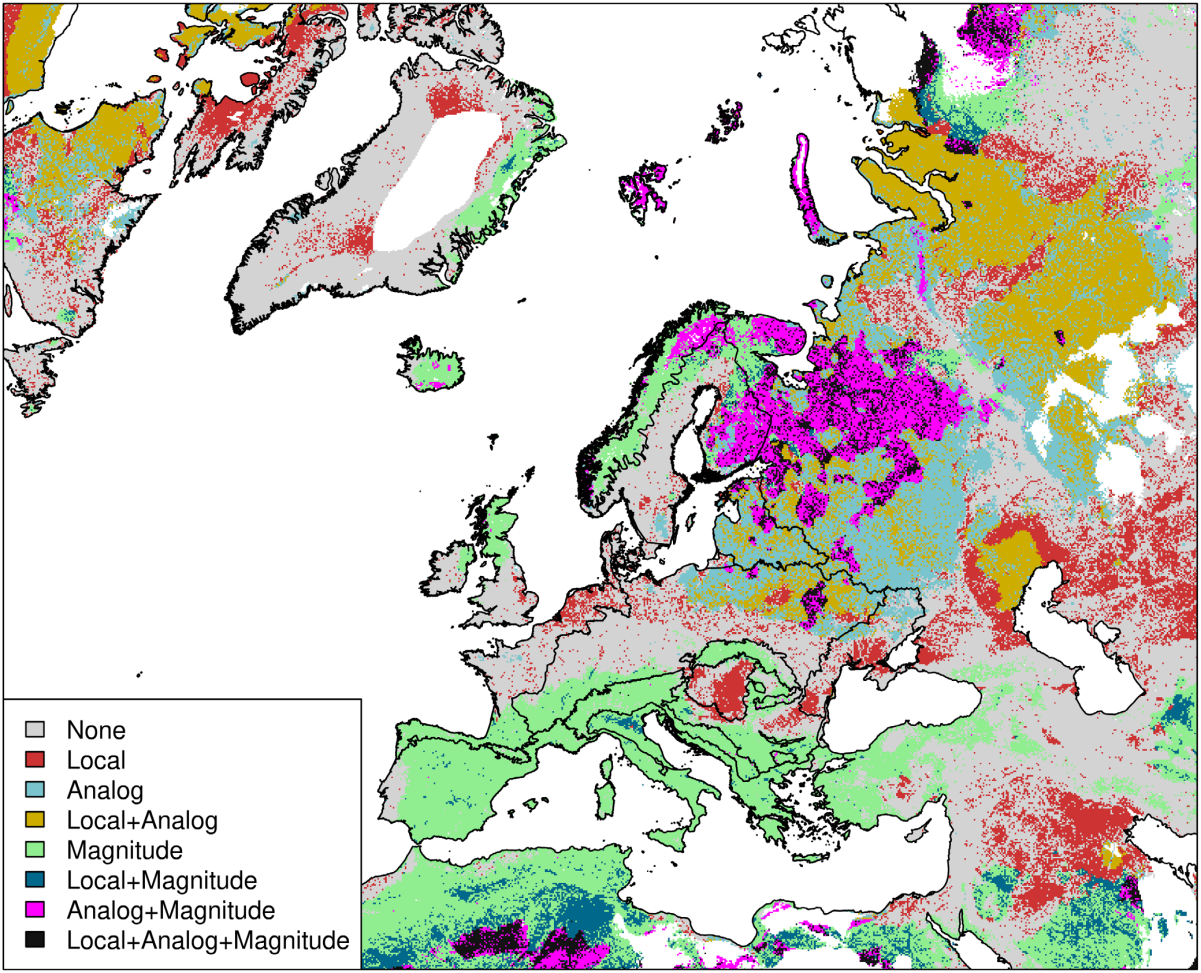
Overlay of the values within the last quartile (higher values) from each climate risk metric for scenario SSP3‐7.0. Areas in the map are color‐coded based on their overlap with hotspots of exposure identified based on different climate metrics (Local, local velocity; Analog, analog velocity; Magnitude). Areas with no analogues within the 1500 km search radius (NA values on land) are shown as blank spots (i.e. central Greenland). Map lines delineate study areas and do not necessarily depict accepted national boundaries.

Areas in North Africa, the eastern parts of the Continental and Boreal regions, and the Baltic stand out as high‐exposure areas in terms of both velocities and magnitude of change under scenario SSP3‐7.0 (Figure 2 and Figure S9). Most areas in the Mediterranean basin and Norway are hotspots of climate magnitude. In contrast, hotspots of local and analog velocity can be found in eastern Europe (Figure 2). We also identified areas where several metrics converged on a likely low risk of climate change exposure (Figure S11). These were especially prevalent in Cyprus, Lebanon, the Syrian coast, and large areas between the Caspian Sea and Persian Gulf. Additionally, parts of the Balkans, particularly in Albania and northern Greece, as well as southern Portugal, exhibited relatively low exposure values for local and climate analog velocity. In northern Europe, some coastal areas of Norway, as well as parts of Iceland and western Greenland, also emerged as potential climate refugia based on low local and climate analog velocity (Figures S9–S11).

We found 61% of PAs are overlapping with hotspots of magnitude or local velocity, including 2.3% areas where the exposure hotspots coincide (i.e., both high magnitude and high local velocity). In comparison, only 2.4% PAs are situated in joint coldspot areas, that is, areas of both low local velocity and low magnitude. When also considering analog velocity, the percentage of PAs falling within hotspot areas of any metric increases to 67%, while the proportion of PAs in shared coldspots (low risk for all metrics) decreases to < 1%.

### 3.2 Climate Risk in PAs vs. Control Sites

**FIGURE 3:**
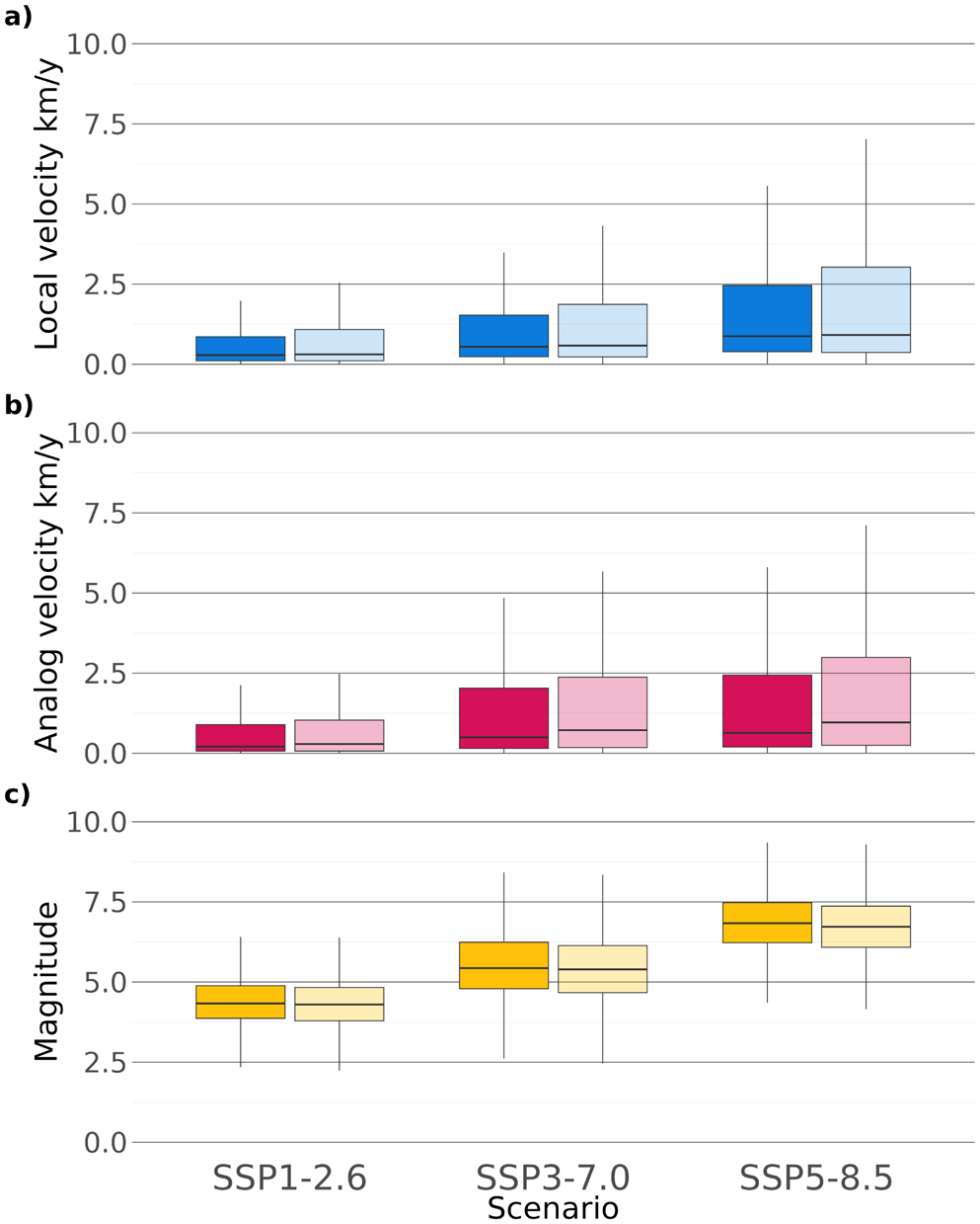
Boxplots showing the range of the three climate metrics inside PAs (lighter color) and in control areas (darker color) under different future climate scenarios: (a) local velocity (blue, absolute sum of the velocities for temperature and precipitation), (b) climate analog velocity (distance‐based method) (magenta), and (c) climate magnitude (yellow). Results for climate analog velocity (distance‐based method) exclude grid cells from matching study areas (Figure S4) for which it was not possible to find future analog climates (0.66%) within the 3 SSP‐RCP scenarios.

Median values of all climatic metrics were extracted for each PA polygon to summarize projected conditions under multiple SSP‐RCP scenarios (Table S3) providing a comprehensive overview for all PAs in Europe. The propensity score matching suggests that PAs are more exposed to climate change compared to unprotected sites, with higher median local and climate analog velocity (distance‐based method), especially under the SSP5‐8.5 scenario (Figure 3 and Figure S13, Tables S6, S7). Conversely, the climate magnitude measure showed an opposite pattern compared to climate velocities (Figure 3, Tables S6, S7), with control areas having higher values of magnitude than PAs. The differences in values of climatic metrics between PAs and control areas were significantly different (Wilcoxon signed‐rank test; Tables S6, S7). Furthermore, our statistical matching process was successful in determining control sites for European PAs: the covariate imbalance was reduced from a mean *C*‐statistic value of 0.744 before the matching to a mean value of 0.535 across all biogeographic regions after matching (Table S5). The standardized mean difference test also performed well for the whole of Europe (Figure S12) since all covariates were adjusted through the matching and reached a good balance within the 0.1 threshold.

The trend for the climatic metrics in the 10 biogeographic regions generally followed the overall European‐wide trend across the scenarios, with greater median values of local and climate analog velocity within PAs compared to control areas, and with climate magnitude showing a very small difference between PAs and controls. In general, the differences in VoCC metrics between PAs and controls were statistically significant at the scale of biogeographic regions. However, there were some exceptions, e.g., for temperature velocity in the Black Sea region under all scenarios, and for climate local velocity in the Mediterranean region under scenario SSP1‐2.6 (Table S4, Table S7). Moreover, PAs in different biogeographic regions showed different levels of climate change exposure (Figure S13).

Under all scenarios, the highest values of local climate velocity occurred in the Pannonian, Boreal, and Steppic biogeographic regions (Figure 1 and Figure S13, Table S7), whereas the Alpine, Mediterranean, and Macaronesia biogeographic regions showed the lowest values. The median value for local velocity of temperature (Figure S14, Table S7) increased under each scenario compared to the baseline period, with the highest values found under the SSP5‐8.5 scenario. Nevertheless, there was a high degree of spatial heterogeneity (Figure S14, Table S7), and the highest values were found again in the Pannonian, Boreal, and Steppic regions.

For precipitation (Figure S15), local velocity was sometimes negative, indicating decreasing precipitation, which can lead to increasingly dry conditions in association with increased temperature. The Pannonian, Steppic, and Black Sea region were estimated to be most affected by drier conditions. The Boreal, Steppic, and Continental regions were overall the most exposed regions based on climate analog velocity values, whereas the Mediterranean and Alpine regions were less exposed. The differences in climate analog velocity between PAs and control regions follow the overall European trend, with higher climate analog velocity values in PAs compared to control regions, except for the Steppic and Pannonian regions (Figure S13, Table S7).

The general trend for climate magnitude was a dramatic increase within PAs across all biogeographic regions when going from the lowest emission scenario to the highest. The Arctic region showed the highest value of climate magnitude across all scenarios, followed by the Alpine and the Mediterranean. In general, control regions showed higher values of magnitude compared to PAs, apart from the Arctic, Boreal, Pannonian, and Steppic regions. Although the magnitude of temperature follows the same pattern, the highest magnitude values for precipitation were found for the PAs in the Arctic and Boreal regions (Figures S14–S15). Overall, the differences in magnitude values between PAs and controls were all statistically significant. However, there were few exceptions; for example, there was no significant difference for climate magnitude in the Black Sea region under any scenario, and no significant difference for the magnitude of mean annual temperature in the Atlantic region under SSP5‐8.5 (Table S7).

The mean residence time across all PAs was 25.5 years, 12.2 years, and 8.0 years for scenarios SSP1‐2.6, SSP3‐7.0, and SSP5‐8.5, respectively. The biogeographic regions with the highest residence time were the Arctic and the Alpine biogeographic regions, followed by the Mediterranean and Macaronesia regions (Figures S16, S17, Table S8). In contrast, the Boreal, Pannonian, and Steppic regions showed the lowest residence times across all scenarios, with low residence times even in the low‐emission scenario (median values < 5 years for SSP 1‐2.6), with some PAs experiencing values below the single year even in the most optimistic SSP‐RCP (Table S3).

### 3.3 Climatic Exposure for Species in the Natura 2000 Network

**FIGURE 4:**
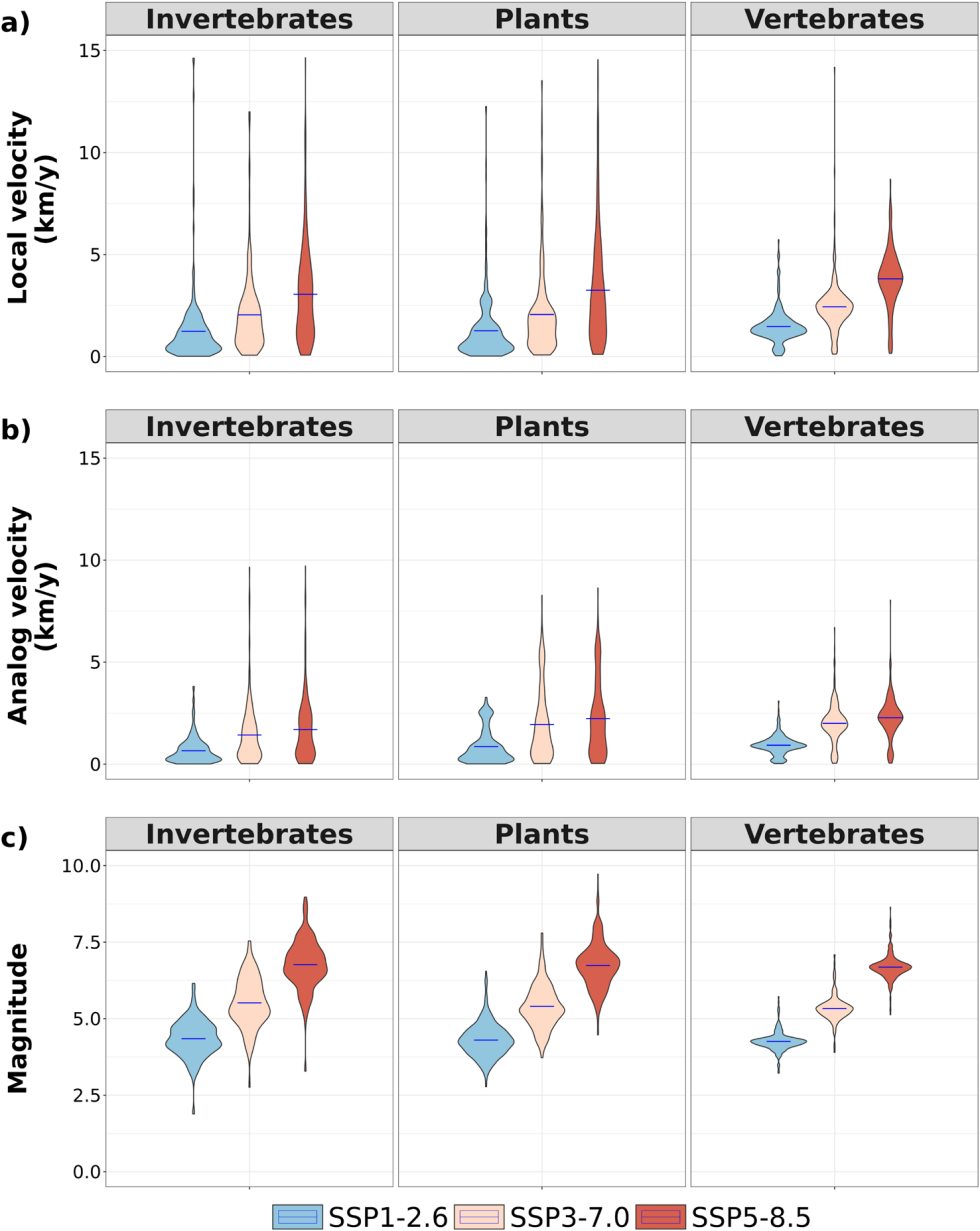
Predicted local velocity (a), analog velocity (b), and climate magnitude (c), for the 1011 threatened species occurring within the Natura 2000 network, presented by main taxonomic group.

The 1011 species identified as threatened by climate change and that occur within the Natura 2000 network showed similar climate change exposure values across the taxonomic groups (Figure 4 and Figure S18, Table S9). For each species, we estimated the median local climate velocity within its Natura 2000 sites of occurrence. We found that the median local and analog velocity of included species' occurrences within Natura 2000 tripled (respectively from 1.18 km/year to 3.45 km/year and from 0.79 km/year to 2.07 km/year) when moving from scenario SSP1‐2.6 to SSP5‐8.5. Similarly, the magnitude increased by ca. 50% (from 4.23 to 6.66).

**FIGURE 5:**
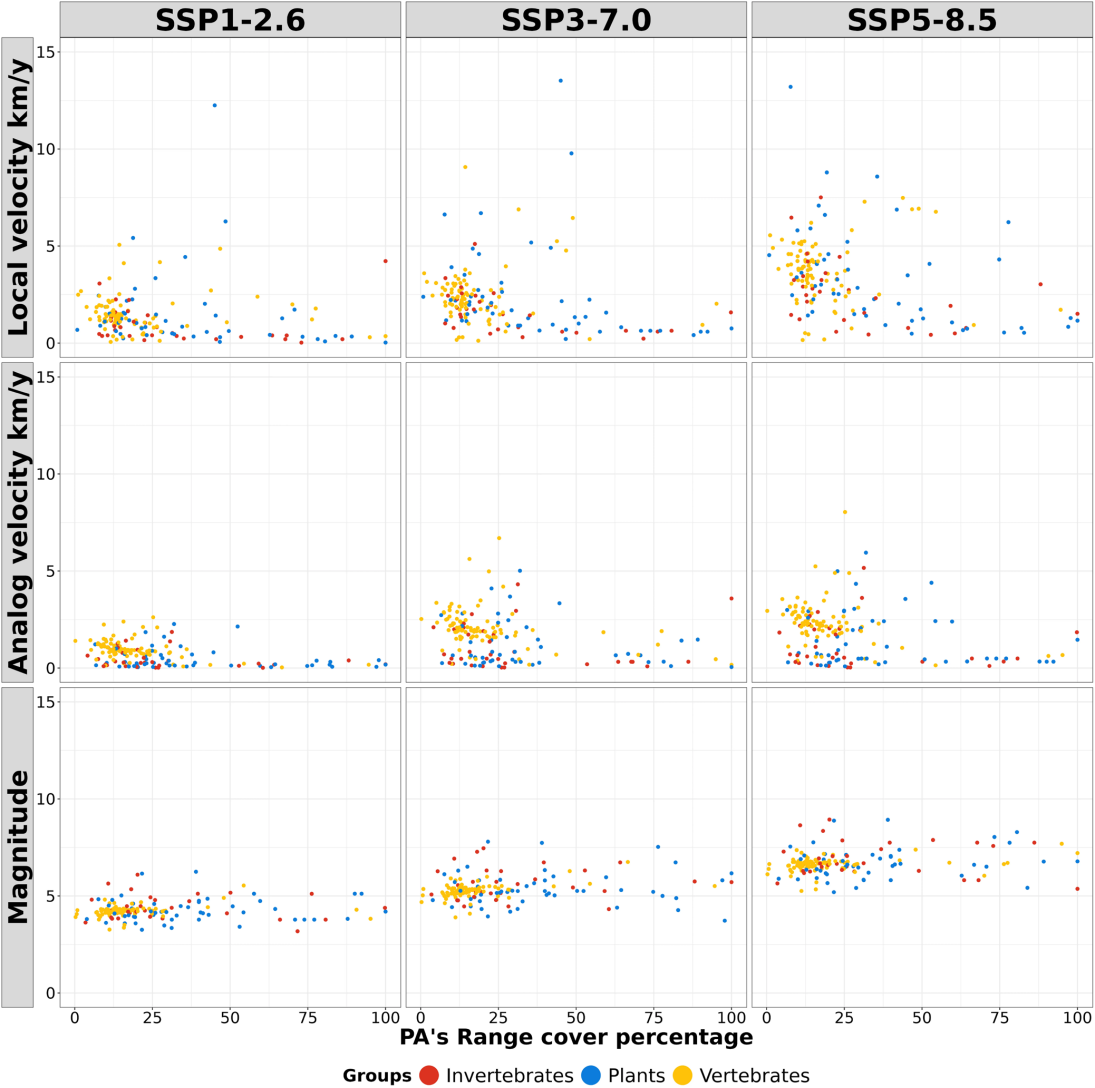
Predicted climate exposure metrics under SSP1‐2.6, SSP3‐7.0, and SSP 5‐8.5 scenarios (*y* axis) compared to the cover fraction of the species distributions within Natura 2000 sites (x axis) for 514 species of invertebrates, plants, and vertebrates.

For the 514 species with detailed spatial distribution information available from the IUCN Red List database, climate exposure within the Natura 2000 network was assessed in relation to the overlap of the species' distribution area with Natura 2000 sites (Figure 5 and Figure S19, Table S10). Climate exposure and distribution area overlap were poorly related, indicating that species with any level of representation within the Natura 2000 network are equally highly exposed to climate change risks (Figure 5 and Figure S19). All species groups showed higher velocities under scenarios SSP3‐7.0 and SSP5‐8.5 compared to SSP1‐2.6 (Figure 5). Eleven tetrapod species had > 50% of their range within Natura 2000 sites, suggesting a high dependence on the Natura 2000 network (Table S11).

## 4 Discussion

Our study provides a multi‐metric and high resolution (1 km^2^) assessment of climate exposure in Europe, based on the latest climate data (those from CMIP 6) and employing a combination of complementary metrics, climate models, and SSP‐RCP scenarios. We found the highest climate exposure in the Continental, Steppic, Boreal, and Pannonian regions (in terms of velocities) and the Mediterranean and Alpine regions (in terms of magnitude). On average, PAs showed lower climate change magnitudes than control sites, but higher local and climate analog velocities. We also found several species facing high climate risk within Natura 2000 sites, including some that are also largely restricted to these sites.

### 4.1 Geographic Patterns of Climate Exposure

Our results for local velocities and climate analog velocities are largely congruent with the forward velocity results of Lai et al. (2022), suggesting that the highest exposure is found in inland Europe within both the Continental and Boreal regions. However, we were additionally able to recognize that the largest climate magnitude changes will occur in the Mediterranean region and in some parts of the Alpine region, but high climate velocities will only sporadically occur in these two regions, probably due to their high degree of spatial heterogeneity. Higher mean climate residence times within the Natura 2000 were projected to occur in the lowland areas of the Pannonian and Steppic regions and extensively across the Boreal region, showing both overlapping highly exposed areas but also some spatial differences, e.g., in the western part of boreal Fennoscandia.

Taken together, our findings highlight that there is substantial geographic variation in the level of climate exposure across areas in Europe, but also large variation depending on the metric considered. This may generate dissimilar pressures depending on species' distributions across Europe. For example, in Northern Europe, contrasting spatial velocity patterns have been projected, with some areas and PAs experiencing high exposure of winter vs. summer‐time temperature velocity, including a full‐scale turnover of current temperature conditions in the PAs (cf. Heikkinen et al. 2020).

In agreement with our results, some earlier studies have suggested that the Natura 2000 network may face major changes in climatic conditions, challenging its long‐term performance in safeguarding valuable biodiversity (Lai et al. 2022; Nila and Hossain 2019; Stagl et al. 2015). For example, Lai et al. (2022) showed that velocity hotspots (i.e., areas facing both substantial (≥ 5 km/year) forward and backward velocities) occur under a high emission scenario across wide, flatter areas of the Boreal and Continental regions and in high‐elevation alpine areas. Coldspots with low (< 0.5 km/year) velocities were found in coastal Mediterranean areas and in low‐elevation areas in the Alpine region. The integrated use of forward and backward velocities by Lai et al. (2022) provided complementary measures, occasionally showing contradicting metrics, that is, spatially varying exposure to different climate change impacts. This, together with our results, motivates the combined use of multiple metrics to reach a holistic understanding of climate change exposure.

Importantly, although the overall results show large variation across the study area and among the different metrics, we also identified certain areas where different dimensions of high climate risks coincide (Figure 2 and Figures S9–S11). Accounting for such joint climate risks is important for conservation planning, as they can help identify elevated exposure and climate refugia to species residing in these areas (Belote et al. 2018; Lai et al. 2022).

When PAs were compared to environmentally similar corresponding areas across Europe, our results revealed statistically significant differences between the median values based on three climate metrics. On average, PAs showed lower climate change magnitudes, but higher local and climate analog velocities compared to unprotected areas. Similar results were obtained by Lai et al. (2022) for the Natura 2000 network based on forward velocity values. However, in our results, these differences were small, although statistically significant due to our very large sample size (1‐km grid cells), suggesting that PAs and control areas are overall facing similar climate change threats.

Overall, our velocity assessments provide crucial information, particularly by allowing the identification of PAs (Table S3) and biomes (Table S7) with the highest or lowest velocities or magnitude into which new conservation activities might be targeted (Hlásny et al. 2021; Lai et al. 2022). For example, we found that, under scenario SSP5‐8.5, one of the peatland dominated PAs (Saaravuoman‐Kuoskisenvuoman soidensuojelualue) located in north Finland was among the PAs most exposed to all metrics (with a values of 3.36 km/year for local velocity, of 7.33 km/year for analog velocity and 6.25 for magnitude) while Hortobágyi Nemzeti Park Igazgatóság, a PA in Hungary, was among the most exposed to local velocity, with a median value of 17.14 km/year. For analog velocity, one of the most exposed PAs was Folgefonna in Norway, showing a value of 12.27 km/year, and for climate magnitude, Mali i Tomorrit, a PA situated in Albania, had a value of 8.39.

For our fourth metric, residence time, there are notable (up to 20‐fold) differences between PAs located in different biogeographic regions, with the Boreal, Pannonian, and Steppic regions showing the lowest and Alpine and Arctic regions the highest residence times (Figures S16, S17, Table S8). This means that in PAs situated in more extreme environments, current climatic conditions will prevail for a longer time than elsewhere. This is in line with earlier studies (see Brito‐Morales et al. 2018 and citations therein) showing a clear linkage between low topographic heterogeneity and shorter residence times, suggesting elevated risks for species in such areas (Heikkinen et al. 2020; Huey et al. 2012; Loarie et al. 2009).

### 4.2 Species Exposure to Different Dimensions of Climate Change

Both climate velocities and the magnitude of changes contribute to reshuffling species distributions and altering ecological interactions (Brito‐Morales et al. 2018; Gaüzère et al. 2023; S. Hoffmann et al. 2019; Lurgi et al. 2012; Williams and Jackson 2007). However, to improve relevance, climate metrics need to be explicitly related to different species.

We found substantial variation in climate exposure among Natura 2000 species included in our analysis, regardless of their taxonomic group. For example, among the 11 tetrapod species with more than 50% of their range within Natura 2000 sites, *Rana pirenaica* exhibited both high local climate velocity and magnitude (7.3 km/year and 6.7, respectively) under scenario SSP‐8.5. In contrast, *Lynx pardinus* showed similarly high magnitude values (6.6) but experienced lower local climate velocity (3.17 km/year). The main differences were found among scenarios, with exposure values up to three times higher based on SSP 5–8.5 compared to SSP 1–2.6 (Figures S18 and S19). This result, however, must be interpreted with caution as different species have different levels of sensitivity and adaptive capacity to climatic change (Foden et al. 2013). For example, diet breadth and habitat specialization determine range shift potential in birds and butterflies, while limited dispersal ability is known to affect amphibians and reptiles (Campbell et al. 2024; Lurgi et al. 2012; McMenamin et al. 2008). Although climate metrics offer a holistic understanding of exposure, it is important to recognize that they encase a simplification that species are locally adapted to current climatic conditions in small spatial locations. In reality, many species have relatively broad climatic tolerances and can thrive in a wider set of climatic conditions than those prevailing in a limited number of spatially defined grid cells.

Given the importance of connectivity and conservation planning, our study focuses on horizontal velocity, as it provides a broad‐scale perspective on species movements. While species are known to also shift to higher elevations in response to climate change (Chan et al. 2024), vertical range shifts are subject to multiple ecological and physiological constraints that may limit their effectiveness as a long‐term adaptation strategy. While we recognize the computational demands of including vertical velocity in large‐scale studies like the one presented here, future studies should attempt to combine horizontal and vertical velocity to assess regional differences and provide more accurate estimates for mountainous regions in Europe.

Nevertheless, the four metrics used in our study represent different aspects of climate change exposure, each with unique implications for biodiversity conservation and PA management (Garcia et al. 2014; Ordonez et al. 2016), facilitating a diverse threat assessment for the species. Areas with low climate velocity, such as mid‐elevation slopes in mountainous regions, likely provide more stable settings. If climate analogs are found in adjacent areas, this can support species in their adaptive responses (Ackerly et al. 2010; Garcia et al. 2014). Local changes in climate conditions—here, climate magnitude exposure—can directly impact species' physiology, morphology, and behavior, leading to demographic changes (Camacho et al. 2024; Khaliq et al. 2014). In particular, species populations that currently occur in conditions close to their climatic tolerance limits and species with specialized climatic requirements or limited adaptive capacity are at higher risk of declines or local extinctions under high exposure to climate magnitude (Araújo et al. 2013; Doucette et al. 2023; A. A. Hoffmann et al. 2013). Our results show the highest levels of climate magnitude exposure occur in the Alpine and Mediterranean regions. In the latter region, changes in temperature together with even modest changes in precipitation can jeopardize populations of species that are less tolerant to extended periods of drought and increased frequency of fires (Batllori et al. 2019). In the uppermost parts of the Alpine region, species are at risk of facing cul‐de‐sacs due to disappearing local climate space (Hamann et al. 2015). This, in combination with future climate analogs located far away, may be detrimental to sessile and slowly dispersing nonvolant species (Dobrowski and Parks 2016; Lai et al. 2022).

In many parts of Europe, projected velocities for temperature exceeded 5 km/year and sporadically even 10 km/year. This pace can be considered critically high as it exceeds the rate of observed geographic shifts even for highly mobile species, such as boreal birds (Heikkinen et al. 2021). For some less mobile species, e.g., dispersal‐limited plant species, high velocities produce substantial obstacles for successful range shifts (Barber et al. 2016). Moreover, even species with higher dispersal capacities, ranging from mammals to bryophytes, have been predicted to lag behind future changes in climate (Lenoir et al. 2020; Schloss et al. 2012; Zanatta et al. 2020). The risks brought by climate change are elevated in the regions where PAs and suitable habitat patches occur as isolated fragments in human‐dominated landscapes (Asamoah et al. 2022). In such areas, higher velocities may increase species range fragmentation and complicate their ability to follow suitable climates (Brito‐Morales et al. 2018; Garcia et al. 2014).

The climate residence times define for how long stable and suitable climate conditions remain in the PAs for species that are dependent on habitats found therein. Residence times were found to be smallest in the lowlands of Continental and Boreal Regions, with median values being lower than two years, suggesting very rapid changes and extensive turnover of local climates in the PAs (Brito‐Morales et al. 2018; Heikkinen et al. 2020; Nila and Hossain 2019). PAs are expected to play a key role as stepping‐stones for species dispersal to new regions (Gillingham and Thomas 2023; Thomas and Gillingham 2015), but species residing in small PAs within human‐dominated flatlands may increasingly lose suitable conditions and face difficulties in shifting across space in pace with climate change (Hannah et al. 2014; Kharouba and Kerr 2010; Parks et al. 2023).

### 4.3 Implications for Developing a Climate‐Wise Trans‐European Nature Network

Biodiversity is becoming increasingly exposed to the impacts of climate change both inside PAs and in the intervening matrix, both globally (S. Hoffmann et al. 2019; Loarie et al. 2009), at continental (Batllori et al. 2017; Belote et al. 2018) and regional scales (Barber et al. 2016; Hamann et al. 2015; Heikkinen et al. 2020; Stagl et al. 2015). Climate risk metrics have become a standard approach for assessing the exposure of biodiversity to climate change (Brito‐Morales et al. 2018). However, our results show that multiple dimensions of climate exposure should be examined to obtain a more holistic picture that can also guide in PA expansion (see also Garcia et al. 2014; Nadeau and Fuller 2015). At the same time, our production of detailed risk metrics for each PA (Table S3) can help managers anticipate future risks and define local climate adaptation strategies.

Despite this, climate change appears to have been largely overlooked in the designation of European PAs, potentially undermining their future conservation value. Thus, simply designating 30% of Europe as “protected” does not guarantee that the biodiversity within these areas will persist in the long term, particularly if those areas happen to be relatively vulnerable to the adverse effects of climate change.

The high climate pressures revealed by our study support the recommendations for integrating climate‐wise considerations into nature conservation and management planning (Brito‐Morales et al. 2018; Nadeau et al. 2015). This is of particular importance when planning the PA expansion in Europe, as part of a Trans‐European Nature Network (Jung et al. 2024; Lai et al. 2022; Rannow and Förster 2014). Many earlier recommendations remain valid also in climate‐wise conservation planning, including: (i) determining key areas where species and habitats are most exposed to vs. better buffered against climate change, (ii) targeting new conservation actions to increase the topographical heterogeneity of PAs and thereby supporting longer climate residence times and extending species holdouts, (iii) increasing landscape permeability and ecological connectivity in areas experiencing high climate velocities to enhance species movements, (iv) tailoring management and restoration in PAs to improve the resilience of species populations to climate change impacts, and (v) establishing new PAs in areas where climate exposure risks are low and the potential for climate refugia is high (Hannah et al. 2014; Mawdsley 2011; Nadeau and Fuller 2015; Thomas and Gillingham 2015).

We argue that the establishment of new PAs should consider levels of climatic stability, considering that less than 3% of existing PAs are located in climatically stable areas (i.e., climatic coldspots). Our results draw attention to the high‐velocity areas in the lowlands of Europe, highlighting the importance of increasing the permeability of the matrix areas and connectivity between existing PAs therein to allow species range shifting. In contrast, areas of high climate magnitude might require adaptive management of species populations and their habitats, combined with reducing pressures from other stressors and possibly a consideration of translocations for some of the most vulnerable species (Mawdsley 2011; Thomas and Gillingham 2015).

A climate‐proof Trans‐European Nature Network should opt for spatial planning that simultaneously prioritizes climatically resilient areas, areas representing a variety of biomes and latitudes, and corridors that provide connectivity between these areas to allow species the best available opportunities to thrive and move along with a changing climate.

## Author Contributions

**Marta Cimatti:** conceptualization, data curation, formal analysis, investigation, methodology, visualization, writing – original draft, writing – review and editing. **Valerio Mezzanotte:** data curation, formal analysis, methodology, software, visualization, writing – original draft, writing – review and editing. **Risto K. Heikkinen:** validation, writing – original draft, writing – review and editing. **Maria H. Hällfors:** validation, writing – original draft, writing – review and editing. **Dirk Nikolaus Karger:** data curation, methodology, software, writing – review and editing. **Moreno Di Marco:** conceptualization, funding acquisition, investigation, methodology, project administration, supervision, validation, writing – original draft, writing – review and editing.

## Conflicts of Interest

The authors declare no conflicts of interest.

## Supporting information


Data S1

Table S3

Table S7

## Data Availability

The data and code that support the findings of this study are openly available in Zenodo at https://zenodo.org/records/14146117. A list of the sources of CMIP6 datasets used in this study can be found in Table S1 and Appendix S1. Baseline and future climatologies were obtained from CHELSA at https://chelsa‐climate.org/downloads/ (version 2.1).
